# The Metric System

**Published:** 1890-11

**Authors:** 


### The Metric System.

It will be of interest to dentists to know that the metric system of wieghts and measures is being brought into prominence again after a lapse of several years since it was the craze. It is now coming to stay, and it is a singular fact and irreconcilable too, that Great Britain and the United States stand alone in their persistent use of heterogeneous standards of weights and measures.

Russia has recently adopted the metric system and it is now in use in all Continental Europe. It is used exclusively in Central and Southern America. The United States legalized the metric system by Congress in 1866, and it is now in use by all scientists and chemists throughout the world, but its disuse generally seems to be due to indolence and not a practical training with the system. The system we use in this country is called the *American system* because no other nation uses it, and is not the same as that used in Great Britain.

At the convention for the seventh decennial revision of the U. S. Pharmacopœia, which met in Washington, D. C., May 7, 1890, the said convention resolved by a large majority that the next edition of the pharmacopœia should have weights and measures expressed in the metric system exclusively. This is the sixth national association which has endorsed the metric system. It behooves the dentist then to become familiar with the system which has but one factor, viz., the *gram*, and this is divided according to the decimal system of enumeration. All the precious metals we have to use will be sold by the *gram*, and small quantities of fluids will be measured as *cubic centimeters.* Each cubic centimeter weighs one gram. An ordinary teaspoon, when full, contains five cubic centimeters. Thus, the unit of weight and measure is identical. In an address sent out to the professions of medicine and pharmacy, and the medical and pharmaceutical colleges of the United States and Canada, for the use of these professions six lines contain all that is necessary to know in order to grasp the situation, as follows:

" 1000 milligrams make one gram.
1000 grams or cubic centimeters make one kilo or liter.
1000 kilos make one ton.
65 milligrams make one grain.
15½ grains make one gram.
31 grams make one ounce Troy."

This unit system does away with having several units as a basis to reckon from. But time and space forbid an extended notice.

Tables for converting customary and metric weights and measures may be obtained from the office of Standard Weights and Measures, T. C. Mendenhall, Supt., U. S. Coast and Geodatic Survey. W.

